# Notes and Comments

**Published:** 1884-04

**Authors:** 


### Notes and Comments.

---

OBSERVATIONS ON HAEMORRHAGIC MALARIAL FFVER.—Dr. J. H. McCaleb, of Pointe Coupee, La, in a lengthy article on this subject in the *New Orleans Medical and Surgical Journal*, has this to say of the treatment:

The hypodermic administration of the sulphate of morphia in this disease is indispensable; on the wakefulness and cerebral irritability during the fever its effects are simply charming; it induces a delightful diaphoresis; relieves almost instantly the irritability of the stomach, controlling vomiting for several hours, thereby allowing time for the absorption of other remedies, and modifies to a most satisfactory extent the nervous effects of quinine. Its administration in the early stages might be preceded by large alkaline drinks, given warm, to induce free emesis, in order to wash out the stomach and correct any acidity that might be present; after free emesis induced in this way, a hypodermic injection of from one-sixth to one-third of a grain of the sulphate of morphia (Magendie's solution), never has failed, in the writer's experience, to afford immediate relief from the excessive jactitation and vomiting, placing the patient in a most comfortable condition.

The hemorrhage of this fever is purely the local expression of a constitutional affection, and the hemæturia is but an effort of the kidneys to eliminate a *materies morbi* from the blood, and, if the little that is absolutely known of the pathology of this disease be correct, diuretics, as advised by some, are clearly contraindicated. The only channel to which we can appeal with reliance for the elimination of effete matters is the intestinal tract. and calomel is the cathartic that can be employed with certainty for that purpose; by stimulating the secernents and exhalants it corrects morbid secretion, relieves an engorged portal circulation by pouring out bile from a congested liver, and to a large degree relieves the irritability of the stomach. Given in large doses, frequently repeated, five or ten grains every two hours, there is no probability of ever producing its constitutional effects, a circumstance by no means to be desired. If its cathartic effects are not sufficiently prompt, mild saline cathartics should be administered frequently in small doses, or large saline enemas might be resorted to. No fear need be apprehended from hypercatharsis, the morphia subcutaneously obviates such tendency; and the fact is, the more copious the evacuations the sooner the patient is permanently relieved, and I have never seen any but the most gratifying results follow free catharsis continued almost to a diarrhœa. A fly blister applied over stomach and liver does much good; it seems to break up a train of morbid associations by relieving congestion of the organs over which it is applied.

The administration of quinia should be commenced as soon as possible. About one or two hours after the second dose of calomel has been given, and the nervous system has been calmed by morphia, five or ten grains may be given in capsule every two or three hours, until twenty-five or thirty grains have been taken; if its administration in this way should induce nausea, and the vomiting should become too frequent to allow time for its absorption, it should be given hypodermically. Twenty-five grains of the sulphate of quinia are added slowly to a concentrated solution of tartaric acid (fʒ) and the quinia is completely dissolved. Ten minims should be given every two or three hours. The hypodermic needle being driven deep into the muscle, there is but the slightest possibility of an abscess resulting. Under all circumstances the patient should be kept quiet as possible—during the administration of the quinia particularly—and should he become restless, small hypodermic injections of morphia give instant relief.

This is purely a malarial disease, and *the specific* for all forms of malarial toxæmia is the only remedy that can be rationally employed as a curative agent, but the writer has witnessed the most *disastrous consequences resulting* where the administration of quinia has not been attended with the precautions to obviate its bad effects, which has been the endeavor of this article to elucidate.

Soon after copious evacuations have been obtained from the intestinal canal, and the fever abates and vomiting ceases, which rarely fails to be the case, although a certain amount of nausea may continue for some time, small doses of beef-tea, or milk, combined with a little brandy, may be ventured upon at intervals of one or two hours, or oftener if the stomach retains it well. After the stomach is able to receive food the tinct. ferri chloridi, in ten to thirty drop doses, considerably diluted, and given after eating, three or four times a day, rapidly restores strength and color. The tinc. cinchonæ comp. is also highly indicated, given before eating and continued for several days during convalescence.

As convalescence approaches, the fever ceases, the urine becomes clear, and the skin and conjunctivæ lose completely the intense, yellow cast. Nausea is about the most lingering of all bad symptoms, but as the patient regains appetite convalescence becomes rapid.

---

POISONOUS SNAKES AND THEIR VENOM.—In the paper read by Sir Joseph Fayrer at a recent meeting of the Medical Society of London, all that is known with regard to the effects of snake venom on living creatures, as well as the present aspect of treatment of the poisoned individual was fully and ably discussed. The importance of the subject is attested by the fact that in India, according to official statistics, the deaths by snake-bite amount to an average of 20,000 persons per annum, and by the further and very deplorable fact that, notwithstanding every means that has from time to time been tried as an antidote against the poison of these reptiles, death as surely follows the bite of a full-grown and strong snake endowed with poison glands and fangs as it ever has done, even from the most ancient times recorded in history. In comparison with this fact all other relations to the study of *thanatophidia* are secondary in importance.

No doubt, a ready and convenient method of classifying Indian snakes is to divide them into viperiform and colubriform. The general aspect of a representative specimen of each of these is sufficiently characterized—*i.e.*, to be at once recognized even by a non-scientific observer, and when it becomes practicable to examine the jaws of these reptiles, either during life or after death, the presence or absence of poison fangs supplies the very simple and conclusive diagnostic; there is also the easily remembered fact that all viperidæ are venomous. With regard to the colubriform, however, not only does the class comprise several kinds, the bite of any member of which is extremely venomous, as also several that are innocuous, but in general outward appearance the characters so closely resemble each other that, except perhaps a trained ophiologist, no one else coming suddenly—in the jungle or arid plain—upon a snake some six feet long and upwards, could at once determine whether it was an innocuous or venomous colubriform reptile. In order to extend knowledge on this point the Government of Madras, some years ago, sanctioned the publication of well-executed prints representing the several orders of snakes; these prints were circulated throughout the Presidency, but it is feared that the classes of natives most liable to be bitten by snakes benefit little, or not at all, thereby—namely, the Ryots, or agricultural laborers, and the poorer classes generally, who live and sleep in fragile huts, the floor of which is level with the ground, or on low bedsteads called charpoys in the open air. It is usually said, and apparently with truth, that whereas in the fields the greater number of accidents occur from the Daboia, a member of the viperidæ, the majority of persons who are bitten during sleep are so by either the Cobra, or Bungarus (Krite). As a rule, the position of the bite is in a measure characteristic of the kind of snake inflicting the injury; the vipers scarcely raise the head when they bite, hence the majority of wounds inflicted by them are no higher up the limb than the dorsum of the foot, or the ankle; the elapidæ, on the other hand, raise the head considerably when they bite, the extent to which they are able to do so being equal to one-third of the length of the reptile. Fortunately, this habit of raising the head often affords a person a very ready means of averting the danger threatened, and destroying the animal, as the vertebræ are very readily broken when the creature is in its characteristic attitude of offense, by a smartly delivered horizontal blow with a stick or small cane. In the case of the terrible Hamadryad of Burmah, the "hill cobra" of the Madras Presidency, a reptile of full grown size, twelve feet in length, its head erect to the proportionate height already indicated, a good deal of resolution is required to stand and deliver the necessary blow. The latter snake, like its race in general, flees from man as a general rule; during the rainy season, however, that is, during their breeding season, it would appear that this rule is by no means without its exceptions. The efficiency as a penetrating and injecting instrument of the poison fang is of three degrees of completeness, increasing from the hydrophida through the elapidæ to the viperidæ in which it attains its most perfect condition for dealing death. The physical and chemical characters of the poison of different sub-divisions of venomous snakes vary, and so do also the physiological effects severally produced by them, although, in regard to each kind, the particular phenomena are similar in themselves throughout nearly the whole range of warm and cold-blooded animals, poisonous snakes alone excepted; in them, the poison, whether of themselves or individuals of the same or of altogether different genera or species, has no effect whatever. Geographical position, even in the same country, modifies the degree of virulence of snake-poison, and there is the further difference observed in phenomena caused by the same genus of venomous snake, according as to whether the reptile belongs to Asia, Africa, America, or Australia. The further peculiarity was noticed, that whereas the venom of some kinds of snakes, more especially the Crotaline, resists a temperature equal to that of boiling water, an exception to the rule occurs in members of the same order, namely, that of the Crotalus adamanteus, it seems to be destroyed by a temperature of 175° F. The poison of all kinds of thanatophidia appears to be destroyed when treated in the chemical laboratory with the various preparations of bromine, iodine, and permanganate of potass. In actual experiment, however, each and every such reagent has been powerless to arrest, or even modify, the power of snake poison when it has been fairly injected into the tissues of a living animal. Persons who have witnessed cases of snake bite in India are familiar with the difference between the effects of the Cobra poison and that of the Daboia—in the former the coagulability of the blood remains; in the latter, it is destroyed, as indicated by the percolation of serum from the wound itself, and from mucous membranes of the individual bitten. Another remarkable circumstance connected with snake poisoning is that venomous properties are by it communicated to the blood of the animal bitten, and that these properties become reproduced through a series of three successions of animals into which blood of this nature has been injected.

Among numerous other points of great importance brought out by Sir Joseph Fayrer are the facts that snake poison may be absorbed by mucous membranes and produce its effects; also that as a consequence, great danger attaches to the practice, so frequently recommended by writers on this subject, of applying the mouth to a snake wound and sucking the poison therefrom. Very grave doubts are also raised against the belief hitherto entertained that the poison is nnocuous when received into the stomach. With regard to treatment, he observes that "there must be a quantity (of the poison), however small, which, though dangerous, is not of necessity fatal; and in such cases we may influence by treatment, and save life in some." Beyond this, neither he nor any other person practically acquainted with the subject holds out any hope whatever of an "antidote" against snake poison being found, and it is well fully to recognize the conclusion thus arrived at after long continued investigation. The same conclusion has over and over again obtained confirmation. In the case of the great mass of the people in India most liable to be bitten by snakes, their innate apathy, added to their simple submission to *Kismet*, or faith, lead them to delay application for treatment until the time when treatment of any kind could possibly be of the slightest use, has long passed. When, therefore, we hear of recoveries having taken place, the true explanation lies in one of three circumstances - either the snake has been exhausted, or harmless, or it has bitten imperfectly. Whatever other differences there may be in results obtained by different investigators, there is unity of opinion that "the idea of finding a physiological antidote for snake poison is a Utopia."—*Medical Press and Circular.*

---

A STRANGE CASE.—The Swedish physician, Santesson, relates in *Hieayg* that he was called to a young man of twenty-five with an abscess in the left iliac region. Examining him M. Santesson found the right side of the scrotum empty, and in the left side a hard, round body of the size of a musket ball, covered by the skin, but no trace of cord or testicle. A linear cicatrix on each side of the median line made him suspect castration. On interrogating his patient he learned that the young man, when seventeen or eighteen years old, had obeyed the Scriptural direction, If your right eye offends you, pluck it out, and had, with a planing iron, made an incision in the skin of the scrotum, extracted the testicle, and cut the cord on both sides. Local applications of cold water arrested the hemorrhage, and he recovered in about three weeks. Certainly it seemed to him disagreeable to live with an empty scrotum, and fearing that he would gain heaven later than he thought, he decided upon a new operation. With the chisel he made an incision in the scrotum on the left side, and introduced into the wound a glass ball, which served as a testicle, and relieved his disagreeable void. The wound healed slowly. M. Santesson extracted the ball, and presented it to the Society of Medicine of Stockholm.—*Boston Medical and Surgical Journal.*

---

DR. J. MARION SIMS is said to have left ready for publication a story of Revolutionary times, entitled "Lydia McKay and Colonel Tarleton."

A CASE OF MONSTROSITY.—A CHILD WITH A DOG'S HEAD.—Dr. C. Hard, of Ottawa, Ill., reports the following case: A German woman was delivered of a very large female child, weighing fourteen and a half pounds. The body was well formed and perfect but the head was almost the exact counterpart of a bull pup, and was the most hideous monstrosity I ever saw. The eyes were high up on the forehead, large and round, and protuberant, and were two and a half inches apart; no eyebrows. The nose was long and flat, and continued to the mouth, with wide open nostrils; the distance from forehead to nose and mouth $3\frac{1}{2}$ inches The ears were small and long, standing out from the sides of the head one inch, and looked like the cropped ears of a stable dog; they were $3\frac{1}{2}$ inches apart. Back of the ears was a single tuft of coarse reddish hair, about one inch long. There was no back part to the head; or rather, no bones, but a simple sac containing a soft, semi-fluid mass. Taking the whole face together, the resemblance to a dog was most striking, and was at once remarked upon by all present, some of whom were anxious that I should despatch it at once, but I allayed their fears by assuring them that the child would not live. It did live four hours, giving out faint moans from time to time.

Owing to the sensitiveness of the parents I was unable to procure the head, even for the purpose of making a cast. The husband, upon questioning the mother, who does not speak English, gave me the following statement: Her father kept a large dog chained to his kennel, and during the early part of her pregnancy, she was in the habit of visiting at his house. While going past the dog one evening he suddenly sprang at her, barking furiously, and frightened her almost out of her senses, and she had labored under the impression ever since that her child would be marked. Such is their story, and I give it for what it is worth. The parents are both well-formed, and have one child two years old, also well-formed and bright, and there were no ties of consanguinity between them. What caused the monstrosity?—*Chicago Medical Journal and Examiner.*

---

TREATMENT OF EARACHE.—It is said that by the following simple method almost instant relief of earache is afforded: Put five drops of chloroform on a little cotton or wool in the bowl of a clay pipe, then blow the vapor through the stem into the aching ear.—*Weekly Drug News.*

A NEW METHOD OF ADMINISTERING QUININE TO CHILDREN.—Dr. F. E. Daniel, of Fort Worth, Texas, suggests the following method, which he has practiced with complete success: Press the powder into the smallest bulk; drop a half teaspoonful of the thick, tenacious part of the white of an egg, place the powder on it carefully, and cover it up with another drop, so as to envelop the powder entirely, without letting it come in contact with the sides or bottom of the spoon. If carefully done, it can be most satisfactorily given. Assure the child, if it be a child, that the dose is not bitter; get its confidence if you can, and you will be surprised and gratified at the ease with which it is swallowed, without tasting it; and it is generally retained by the most irritable and sensitive stomach.

There is another positive advantage of this mode of administration over any yet proposed, in addition to the admirable property just mentioned: Being pure albumen, and therefore easily digested and highly nutritious, it is admirably adapted to the condition just mentioned, where there is a positive loathing of food, and the stomach refuses almost anything put into it. We believe it possesses anti-emetic properties, being cooling and soothing to the congested mucous membrane, and acting as we believe mucilaginous substances do, in similar conditions. No doubt many are familiar with this plan; nevertheless, there are doubtless many, also, who are not. At any rate, those of our readers who have never tried it are recommended to do so, and we believe they will never have occasion to regret it. It is infinitely superior to any method ever practiced by ourselves, and we feel that we would as soon be without the resource of quinine itself, as without this means of giving it to sick children. —*Courier-Record of Medicine.*

---

COTTON CANDLE-WICK TAMPONS (FOSTER) —The time-honored cotton ball tampon has, as a dressing in my practice, been to a great extent supplanted by Foster's candle-wick dressing. The wicks are made into a rope, containing ten or twelve strands, from which a piece is cut off the length required; to one end a string is attached to facilitate its removal, while the other end can be moistened with whatever application is to be made. Not the least of the advantages of this dressing is the ease with which it can be applied through a small speculum, and with it the columning and packing can be done to perfection. The facility of its removal is by no means its least recommendation.—*From Report of R. J. Nunn, M. D., in Trans. Med. Association of Georgia.*

THE PROGNOSIS IN CASES OF POST MORTEM WOUNDS.—Dr. W. Thornton Parker, of Morristown, N. J., writes: "The medical journals of this country and Europe have reported during the past year several deaths in the medical profession from post-mortem wounds. Some of these deaths have occurred very soon after the receipt of the injury, others have weakened the system of the victim for years, until some ordinary disease has found him in a state to be easily overcome. I have in mind at present two or three medical men who are suffering constitutionally from post-mortem wounds received several years ago. In looking up the matter in our text-books on surgery, I find too little of information concerning this subject. I supposed that it was generally admitted that the post-mortem wound was injurious to health to such an extent that the virus having once sharply attacked the system, it would be well-nigh impossible to eradicate it. I am well aware that many post-mortem wounds are received, and that little, if any, disturbance seems to have been created by them; but I hold that the system can be, and undoubtedly often is, seriously impaired for life, the natural forces weakened, and the nervous system affected. Hamilton gives this matter very careful consideration, but omits the prognosis in such cases, and so do most of the writers to whose works I have access. The needle seems to be recognized as probably the most dangerous implement inflicting this dreadful wound. My object in sending you this communication is to ask for information from the medical profession on this subject, especially concerning prognosis. During the past week, in conversation with a medical man of high standing, and a man well read in surgery, he informed me that after fifteen years he considered the poison to be so thoroughly eliminated from the system of any one receiving such a wound as to be practically non-existent; and, furthermore, he held that our bodies change so rapidly and continuously that little of the body fifteen years ago remains to-day. . . . Early in 1882 this matter received considerable attention in the columns of the *British Medical Journal*, and one case, at least, was reported where the post-mortem warts had persisted for fifteen years! (See *British Medical Journal*, February 10, 1883.) If the warts can persist that length of time, the poison can continue its injurious effects as long, and even longer, in my opinion, and, as I have reason to suppose, in the opinion of most medical men. I shall be grateful for practical information on this subject, and for reference to works or papers which sustain either view of the prognosis mentioned."—*N. Y. Medical Record.*

